# An Optimized Small Tissue Handling System for Immunohistochemistry and *In Situ* Hybridization

**DOI:** 10.1371/journal.pone.0159991

**Published:** 2016-08-04

**Authors:** Giovanni Anthony, Ju-Ahng Lee

**Affiliations:** Department of Biological and Biomedical Sciences, North Carolina Central University, BBRI 215, 700 George Street, Durham, NC, 27707, United States of America; University of Colorado, Boulder, UNITED STATES

## Abstract

Recent development in 3D printing technology has opened an exciting possibility for manufacturing 3D devices on one’s desktop. We used 3D modeling programs to design 3D models of a tissue-handling system and these models were “printed” in a stereolithography (SLA) 3D printer to create precision histology devices that are particularly useful to handle multiple samples with small dimensions in parallel. Our system has been successfully tested for *in situ* hybridization of zebrafish embryos. Some of the notable features include: (1) A conveniently transferrable chamber with 6 mesh-bottomed wells, each of which can hold dozens of zebrafish embryos. This design allows up to 6 different samples to be treated per chamber. (2) Each chamber sits in a well of a standard 6-well tissue culture plate. Thus, up to 36 different samples can be processed in tandem using a single 6 well plate. (3) Precisely fitting lids prevent solution evaporation and condensation, even at high temperatures for an extended period of time: i.e., overnight riboprobe hybridization. (4) Flat bottom mesh maximizes the consistent treatment of individual tissue samples. (5) A magnet-based lifter was created to handle up to 6 chambers (= 36 samples) in unison. (6) The largely transparent resin aids in convenient visual inspection both with eyes and using a stereomicroscope. (7) Surface engraved labeling enables an accurate tracking of different samples. (8) The dimension of wells and chambers minimizes the required amount of precious reagents. (9) Flexible parametric modeling enables an easy redesign of the 3D models to handle larger or more numerous samples. Precise dimensions of 3D models and demonstration of how we use our devices in whole mount *in situ* hybridization are presented. We also provide detailed information on the modeling software, 3D printing tips, as well as 3D files that can be used with any 3D printer.

## Introduction

The advent of desktop publishing and personal printing devices in the 1980s fundamentally changed the way people create printed documents. Error-intolerant type writing and time-consuming cycles of sending documents to and from the professional printing service were replaced with user-friendly desktop publishing/word-processing and convenient printing from desktop printers. At the present time, equivalent or potentially even more significant changes are being introduced by the introduction of 3D printing technology: the social theorist Jeremy Rifkin predicts personal 3D printing as one of central driving forces for what he refers to as the third industrial revolution [1]. Indeed, early applications of this personal manufacturing technology have already demonstrated great promise in a bewildering array of fields, including rapid prototyping, parts manufacturing, education, robotics, pre-surgery simulation, organ fabrication, prosthetics, apparel design, paleontology, medicinal pill synthesis, material science, and art, to name just a few [2–6]. With the ever-increasing build resolution, some of today’s 3D printers can print out devices with micrometer precision or even nanometer precision [7, 8]. Recently, 3D printing technology was also used to create an orientation device for zebrafish embryos[9, 10]. Another report relevant to the present study demonstrated 3D printed microfluidics devices [11], given that microfluidics devices have been used with live zebrafish embryos [12].

We built a custom tissue handling system with stereolithography-based 3D printing technology. Our system is particularly suited for handling multiple small tissue samples in parallel, using commercially available 6-well tissue culture plates. We have successfully tested this system for whole mount *in situ* hybridization of fixed zebrafish embryos. As this system was designed to be readily modified for fully automatic tissue handling in any XYZ machine, we believe that applications beyond histology could be envisioned according to the end user’s needs.

## Results

### Main Features of The 6-Well Chambers

**Fig 1:**
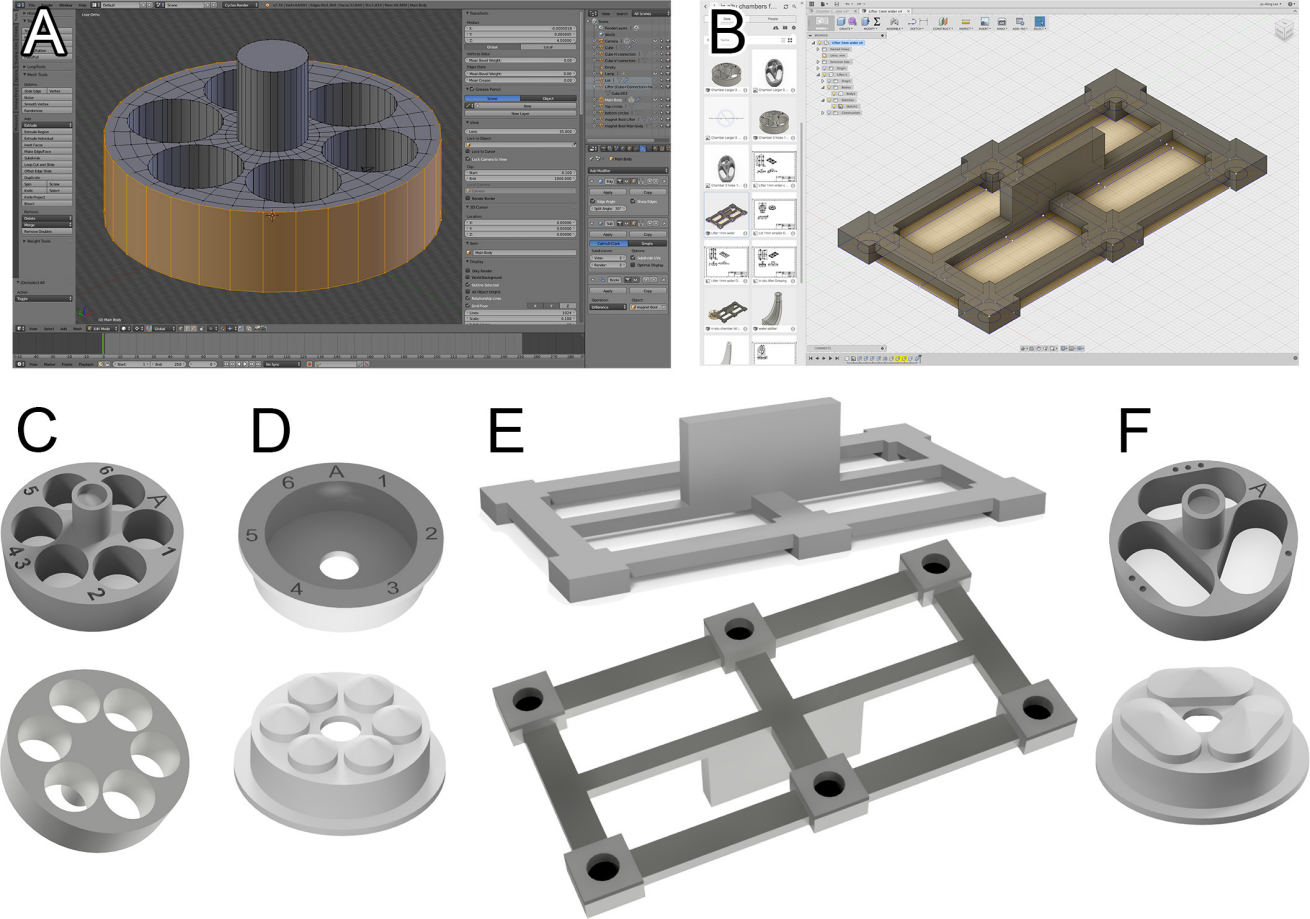
3D modeling programs used in this study and image rendering of the devices. Despite the transparent resin used for actual manufacturing, the images were created with a simulated opaque material for better visualization. Each device was rendered from top and bottom angles. (A) A chamber model shown in Blender 3D. (B) A lifter model shown in Fusion 360. (C) Rendered images of a chamber. Notice that the surface engraved labeling is visible on top of the chamber. A shallow hole for a magnetic disc is found at the top of the cylindrical handle. (D) Rendered images of a lid. Surface engraved labeling is shown on top of the lids. (E) Rendered images of a lifter. 6 holes at the bottom of the lifter would accommodate 5mm x 2mm magnet discs. The rendering of the lifter is not to scale in relation to the renderings of the chamber and lid. (F) Rendered images of a chamber and a lid with larger holes to accommodate more/larger samples.

Our original system contained individually detachable 6 cells for each liftable chamber core (data now shown). However, no significant benefit was obtained from this detachable design. On the contrary, occasional falling of cells made the entire system prone to sample loss. Thus, we altered the design to make the system simpler and more robust. We introduced 6 holes for each chamber. This was a strategic compromise between our desire to process as many samples as possible and the practical ease of tissue handling through small openings (Fig 1C and 1D). We found each well comfortably accommodates more than 3 dozen zebrafish larvae. During our test run, we encountered a problem when multiple different samples were used: namely, easy confusion between samples. To address this issue, we added surface engravings on top of chambers and lids (Fig 1C and 1D). Specifically, by generating 3D model files of 6 different chambers and their lids, each engraved with A to F, we could track each chamber separately. Moreover, separate numbers (1–6) were engraved next to each well. Thus, any well can be easily identified with a combination of a letter (A-F) and a number (1–6): 1A, 3C, 5F, etc. Although the labels were discernable from an oblique angle, it requires effort to clearly read them as the letters are shallow grooves on the transparent body. We could enhance the readability of the labels as follows. A small bit of non-drying modeling blue clay was applied on top of the letter grooves and then cleaned off by smudging with Kimwipes, leaving the grooves filled with the blue clay. A thin layer of superglue was uniformly applied to seal off the blue clay. Each lid-chamber pair was tested for smooth closing and opening: a quick filing with a triangular file was enough to ensure the proper closure of the lid through the cylindrical handle. 3D printer-ready STL files for the 6-well and 3-well chambers with bigger holes are available in S1 File and S2 File, respectively.

### Main Features of The Lids

Unlike IHC, *in situ* hybridization includes a high temperature (~70°C) treatment for a prolonged period of time (overnight probe hybridization). Our initial test showed that if we use a small amount of hybridization solution (~2ml) for each chamber, evaporation of the solution left the tissue dry even if the whole plate was kept in the humid chamber with the plate’s lid closed. Thus, we designed a custom lid that fits our chamber. Our first attempt with a flat surfaced lid could not solve the problem, however, as the evaporated water remained as condensation on the lid. Our next model, equipped with a pointed end facing towards the bottom of the well, successfully prevented the evaporation and tissue from getting dried, as water condensation on the lid readily dripped.

**Fig 2:**
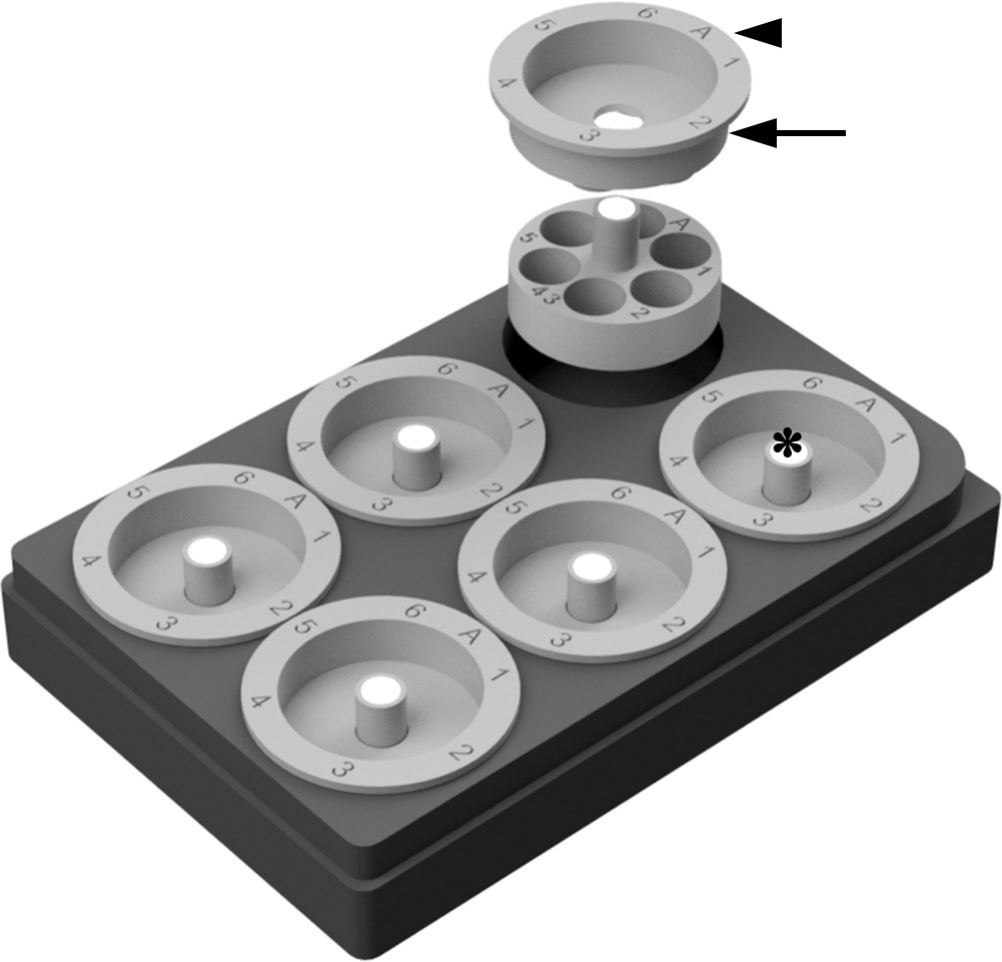
Arrangement of chambers and lids on a 6-well plate. Notice that the flat rim (arrow head) and cylindrical side wall (arrow) of the lid prevent water from evaporating out of and dripping into the well. The rim makes direct contact with the top edge of the plate’s well. The length of the chamber’s handle was designed such that the magnet (asterisk) directly contacts the corresponding magnet of the lifter, even with the circular lid placed on the chamber.

**Fig 3:**
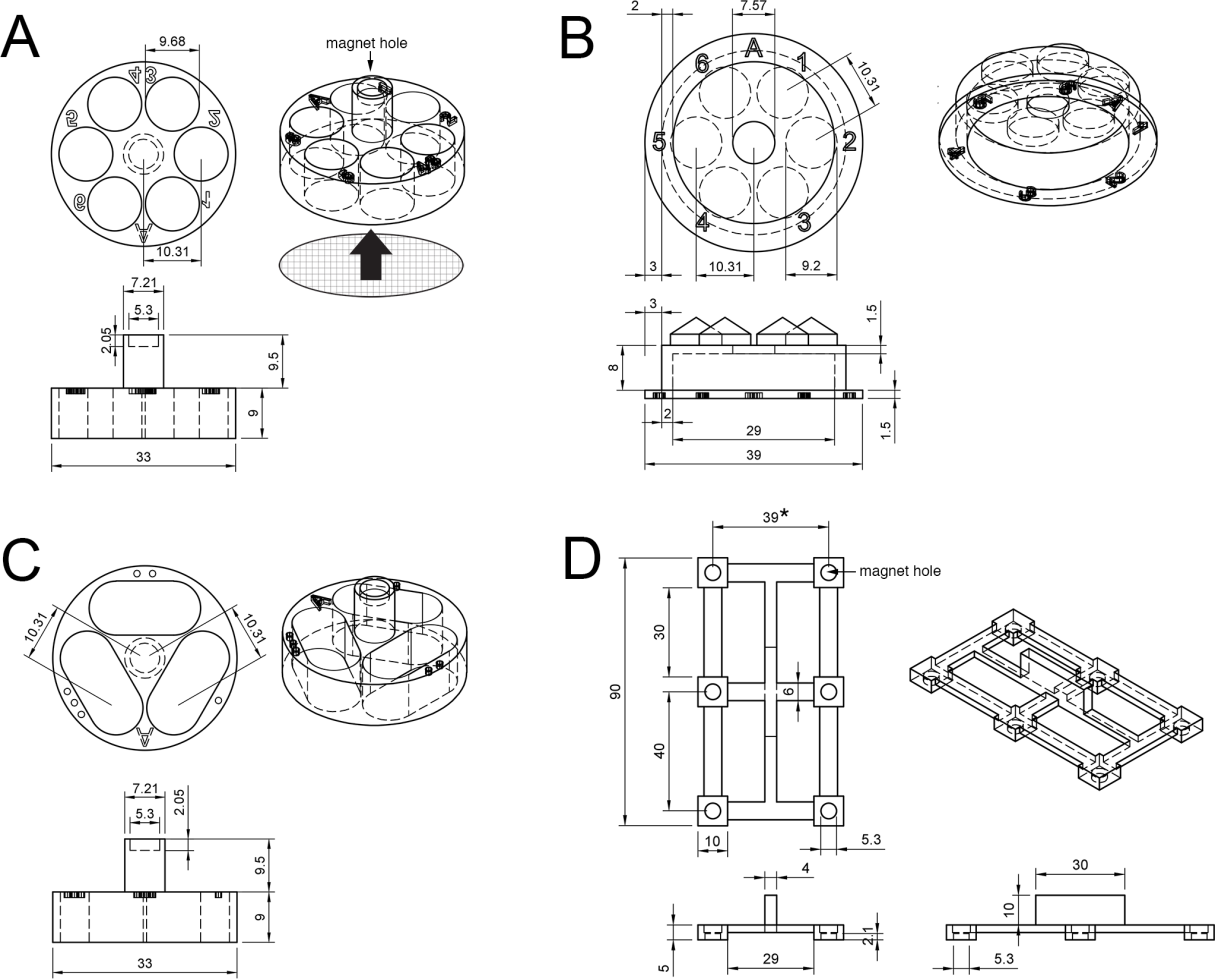
Detailed blueprints for each component. (A) A blueprint for the 6-well chamber. A circularly cut piece of mesh (nylon or stainless) is glued on the bottom of the chamber, as shown with a meshed circle and a large arrow. (B) A blue print for the 6-well lid. Letters (A, 1–6) indicate the surface engraving. (C) A blueprint for the 3-well chamber. (D) A blue print for the lifter. Notice the width of 39mm (asterisk) is 1mm wider than the known dimension (38mm) of the standard 6 well plates (see text). The drawing of the lifter is not to scale in relation to the drawings of the chamber and lid. All units are in mm.

Certain riboprobes for in situ hybridization work better when they are hybridized for an extended period of time, such as 3–4 days (personal observation). We encountered an unexpected problem with such a long incubation. Specifically, a large amount of water vapor from the humid chamber was condensed on the inner side of the plate’s lid and trickled into the wells, which diluted the hybridization solution significantly—more than 100% dilution after 3 day incubation. Thus, we re-designed the lid to include a round side wall and a thin circular rim that seals off the well to prevent accidental water drip (Figs 2 and 3B). With the enhanced lid design, the volume of hybridization solution inside the well remained unchanged. For a long incubation at a high temperature, we found that it is better not to use the 6-well plate’s lid as long as it is kept in the humid chamber. Additionally, a gabled (^ shaped) lid for the humid chamber (or water bath) helps prevent the condensed water from dripping into the plate. 3D printer-ready STL files for the lids for 6-well and 3-well chambers are available in S3 File and S4 File, respectively.

### Magnetic Lifter

A small but functionally important point of our lifter design is noteworthy. Our initial model was built in accordance with the dimension of standard 6 well cell culture plates: the distances between the well centers are 38mm (width) and 40mm (length). However, when we made our lifter to this dimension, magnetically attached chambers were inserted into the 6 well plates with substantial friction. Two plates from different vendors yielded identical results, i.e., significant friction. Upon inspection, we noticed the friction was mainly caused because the distance between the neighboring chambers was slightly too close, only in the direction of width. Since the gluing of magnet discs can cause slight distortion of angles (our observation), we used a completely fluid form of instant adhesive and ensured the leveled adhesion of discs before the glue set. However, even the most careful installation of magnet discs did not solve the friction. Thus, we increased the distance between the neighboring centers of the discs on our lifter from 38mm to 39mm (Fig 3D, asterisk), which solved the problem instantly. To better reduce the potential mal-alignment problem, especially for the full automation—see below, we also reduced the diameter of the chambers and lids from 34mm to 33mm (Fig 3A and 3B).

**Fig 4:**
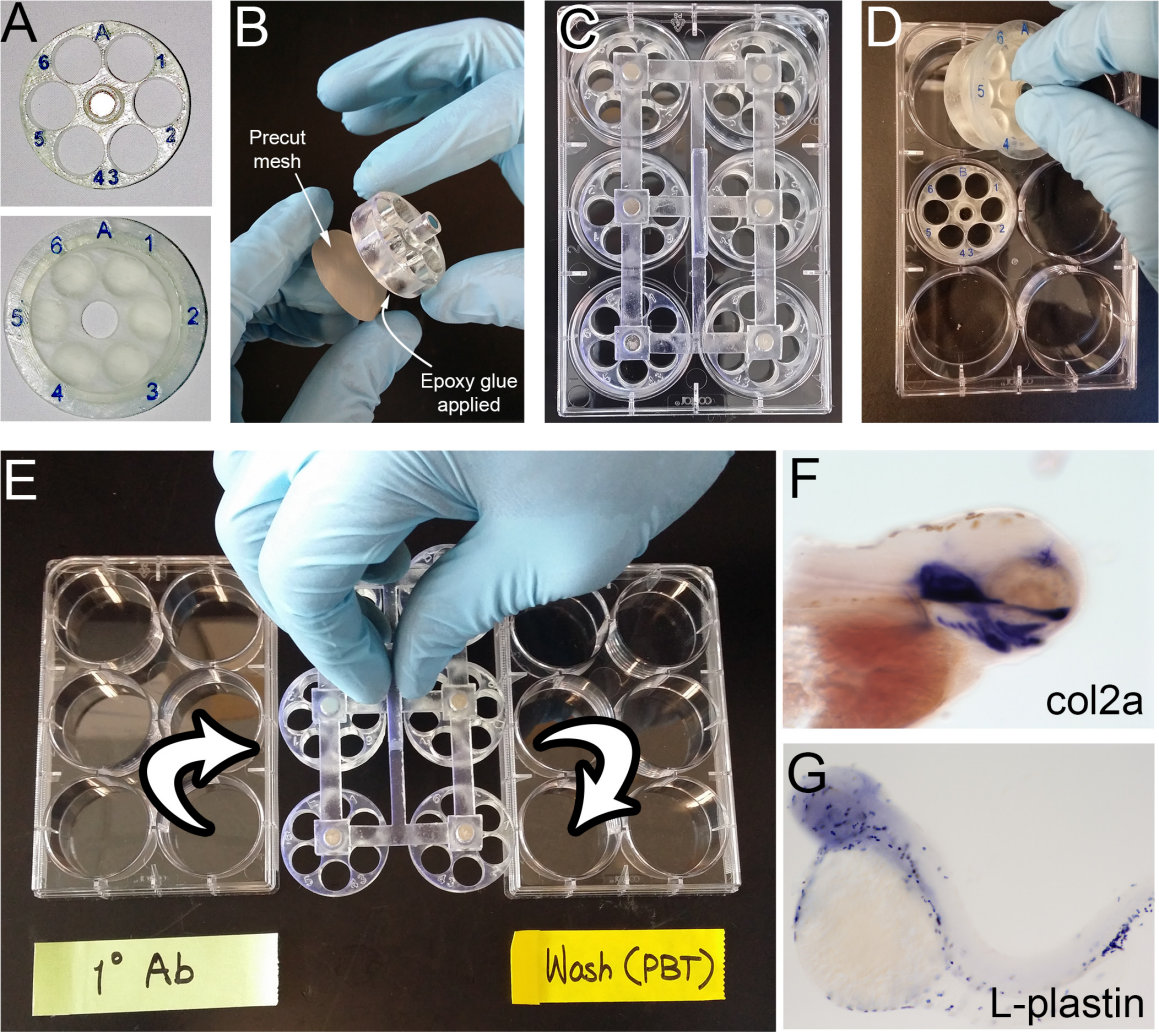
Actual photos of the system and its use for *in situ* hybridization. (A) Enhanced labeling with artist’s modeling clay and coating. (B) Mesh attachment with epoxy glue. (C) A fully loaded plate with a magnetic lifter attached. (D) With only a few samples, individual chambers can be conveniently transferred manually. Chamber A, with its lid closed, was picked up by hand, whereas the chamber B is shown without its lid in pace. (E) 6 chambers (36 samples) are handled simultaneously with a magnetic lifter. (F, G) Examples of whole mount *in situ* hybridization results.

After a few trials of different versions, the height of the chamber’s cylindrical wall was lowered to 9mm to make the tissue handling easy but deep enough for stable transfer of samples through many washing steps (Fig 3A). The length of the handle was decided to make it easy to manually transfer the chamber (Fig 4D), as well as to be magnetically attached to the lifter, even with the chamber’s lid in place. While it is convenient to leave the magnetic lifter attached to the chambers during liquid treatment steps, one can readily detach the lifter by holding three chambers in the 6-well plate with the free hand’s index finger and gently detaching the lifter from the three chambers. One can then hold the remaining three chambers in the same manner while detaching the lifter from them. A 3D printer-ready STL file for the lifter is available in the S5 File.

## Discussion

Previously, attempts have been made to reduce the repetitive liquid handling time common to histology techniques such as immunohistochemistry and *in situ* hybridization. Notable early attempts include the use of plastic “baskets” which were hand-crafted by cutting readily available microcentrifuge tubes and attaching nylon or metal mesh at the bottom of the baskets [13, 14]. The benefit from these early hand-made devices was already apparent. First, simple transfer of baskets by forceps significantly saved time compared with repeated liquid exchange by pipetting. Secondly, the flat bottom mesh maximized the even exposure of tissue to the reagents, in contrast to the typical incubation at the bottom tip of the vertically positioned microcentrifuge tubes. However, these approaches left room for improvement. In particular, manual transfer with forceps of individual baskets still requires substantial time for each sample handling and is also vulnerable to accidental drop of the baskets. Moreover, cutting the hard plastic tubes into open cylinders involves sizable safety risk, whether the tubes are cut by cutters, blades, or saw. Our 3D printing-mediated devices offer benefits in these aspects. Specifically, our approach does not include risky plastic cutting. Most significantly, by assembling multiple holes into a chamber, even manual transfer of chambers without the lifter still significantly cuts down the time for repetitive steps, with much lower risk of accidental slippage. The time-saving achieved by our system is maximized when the magnet-mediated lifter is used to simultaneously handle up to six chambers (6x6 = 36 samples). Moreover, we believe that our system can be readily modified to be used with any standard, programmable XYZ robotic arms.

Though our laboratory seldom needs chambers with larger holes, the need for handling bigger tissue or more number of small tissues will necessitate the design change. Such changes in the pre-made 3D models may require substantial remodeling in mesh-based 3D modelers. However, Fusion 360’s parametric modeling approach provided a powerful means to visit an old version in history and generate multiple different models without re-modeling the subsequent steps. For example, we could rapidly generate a modified 3D model of a chamber with 3 larger holes, which took just a few minutes (Fig 1D). Moreover, as a tool specifically built for engineering CAD, Fusion 360 provides various forms of presentation of the finished models, including standard 3D rendering and interactive 2D blueprints as shown in Figs 1 and 3.

As we increased the number of samples for one round of experiment, we needed to develop a labeling system to distinguish different samples. Thus, we incorporated surface engraving on the top of both chambers and lids. We created 6 different 3D models of chamber-lid pairs. Thus, individual chambers and lids can be readily identified with a combination of A-F and 1–6: 1A, 3C, 5F, etc (Fig 1).

We tested magnet discs in two different sizes (4mm x 3mm and 5mm x 2mm), both of which were made of neodymium. For the final products, 5mm x 2mm magnet discs were used for more surface area to achieve better adhesion. Our decision to use magnets for lifting was based on our future plan to automate the entire process. We envision that the rectangular handle of the lifter designed for manual handling could be readily changed to adopt a bigger magnet (or a grid of magnets for better precision) and the corresponding magnetic frame could be readily printed out to be equipped on any standard programmable XYZ robotic arm. This would enable a full automation of the tissue handling.

SLA 3D printers, despite their distinct advantage of high resolution, have some drawbacks. Most notable are the lengthy printing time and rather involved post-print procedures. Some of the most recent innovations on SLA printing technologies markedly reduced the printing time [15]. However, the complete removal of sticky resin from the printed parts still requires the use of organic chemicals such as pure isopropyl alcohol, which is not only inconvenient and hazardous but also creates a problem of safe disposal of solvent containing the dissolved resin, particularly at home. We disposed leftover resin only after it is completely cured to become a hard block. Moreover, our access to chemical fume hood gave us an easy and safe means of disposal of the used isopropyl alcohol: simple evaporation of the isopropyl alcohol in the hood and curing of the remaining resin.

During our *in situ* hybridization test, we encountered one unexpected caveat, which limits the use of our devices, at least as produced in the way described here. At the end of color reaction in widely used digoxigenin-alkaline phosphatase *in situ* hybridization, tissues are sometimes cleared in organic solutions such as the mixture of benzyl benzoate and benzyl alcohol [16]. This solvent mix, however, melted the surface of the cured resin that we used for this work, leaving some of the fine structures disappeared: parts of the thin walls between wells were melted away. We have not tested if the same solvent mix dissolves other printing materials commonly used in other types of 3D printers. This result demonstrates the need for developing diverse 3D printer resins and for careful evaluation of particular 3D printing materials for specific usage. Encouraging in this regard is the current trend of rapid development of diverse functional resins, including the resins whose printout is either flexible or burns out cleanly without residue, ideal as a mold material (http://formlabs.com/company/press/formlabs-announces-new-suite-functional-resins/).

In conclusion, we developed a 3D-printed tissue handling system that provides numerous advantages such as significant time-saving, superior tissue preservation, minimal sample-to-sample variations, and readily modifiable design flexibility. The STL files of the 3D models are available as supporting information (S1–S5 Files), which can be downloaded and used in any standard 3D printers (not limited to SLA printers).

## Materials and Methods

### 3d Printer

We used Form1+ SLA 3D printer from FormLabs (http://formlabs.com/products/3d-printers/form-1-plus/). PreForm is an accompanied software that we used on iMac desktop computers (Apple Inc.) to send the 3D models to the Form1+ printer. We used the following settings: Clear Resin version 2, 0.05mm. It took about 5 hours to print 9 chambers/lids with the z axis resolution of 0.05mm and about 2.5 hours with the 0.1mm z resolution.

### 3d Modeling Softwares

For 3D modeling, we first used Blender 3D, a powerful and comprehensive open source 3D software package, widely used by motion graphics professionals and artists. Despite rather limited usage for precision CAD, this software could be readily used for our modeling (Fig 1A). Eventually, however, we switched our main modeling software to Fusion 360 (Autodesk Inc.) as this cloud-based software was primarily built for precision CAD and CAM applications (Fig 1B). Fusion 360 can be used as a free software with a 3-year free license for personal or educational purposes.

### Resins for 3d Printing

We tested two different resins built by FormLabs: grey version 2 and clear version 2. Although both resins were compatible for our purpose, we decided to use clear resin due to its transparency that helps visually inspect samples within the chambers.

### Post-Print Processing

For post-print cleaning, we used 99.5% isopropyl alcohol (VWR, BDH1133-4LP). Specifically, upon the completion of printing, the printed parts were carefully detached from the build platform and rinsed in the post-rinse tubs provided with the printer. For the first rinse, the tub with isopropyl alcohol and parts was vigorously shaken with its lid closed for two minutes. If printed parts include small crevices, we also used disposable plastic transfer pipettes (Fisher Scientific, 13-711-9D) to clear remaining uncured resin from the crevices with strong jets of isopropyl alcohol. Once the first rinse was finished, parts were transferred to the second tub with fresh isopropyl alcohol and rinsed for about 10 minutes on a wave motion shaker or by manual shaking. Used isopropyl alcohol from the first and second tubs was filtered with paper coffee filters and stored in separate bottles. Filtered alcohol from the first rinse was re-used up to 3 times and replaced with the alcohol from the second rinse. As the uncured resin for Form1 printers has been recently shown to be toxic in the zebrafish toxicity assay [17], we disposed all unused resin in readily available transparent plastic containers and thoroughly cured the resin in sunlight. In addition, the resin-containing used isopropyl alcohol was kept in a well-ventilated chemical fume hood overnight to evaporate the alcohol, leaving the dissolved resin at the bottom, which was subsequently cured in sunlight for disposal.

The rinsed parts were kept next to the sun-lit window for at least two days and the scaffolding supports were carefully removed using the provided flush cutters. Remaining support marks were removed and the area was smoothened using a triangular/three-square file (Grainger, 24N391).

### Magnet Disks and Tissue-Collecting Mesh

5mmx2mm neodymium magnet discs (www.magnets365.com) were used for lifting chambers. To ensure the consistent angle of the glued magnets, we used liquid form of superglue (also known as krazy glue) to attach the magnet discs into the magnet holes. Before the glue sets, each magnet was evenly positioned to the casing.

For the tissue-collecting mesh, we used nylon mesh with 210μm opening size (Small parts, CMN-0210-C), 300μm opening size (Ted Pella, Inc. 41-X105), or stainless cloth with varied opening size (Grainger wire cloth). Circular meshes were initially made by drawing a circle on the mesh along an unused chamber and by cutting with a pair of scissors. We eventually selected 300μm opening mesh and cut out the circles using a laser cutter.

For chemical resistance and stable adhesion, we used epoxy adhesive (J-B Weld, 50112) to attach the mesh to the bottom of the chamber. Flat wooden coffee stirrers were used to apply the adhesive evenly and accurately, before the chamber was pressed down to the pre-cut circular mesh. It is important to keep the epoxy glue layer consistently thin in order to keep the height of the chambers consistent.

### Support Editing

When resin-based 3D printers print, the objects are connected to the base platform by numerous support structures that need to be removed after the printing is finished. As the contact point of support and the main object slightly distorts the fine surface geometry, delicate surfaces that need precise geometry such as small engraved letters should avoid direct contact with supports. In the PreForm software, support positions can be edited: Supports > Edit Selected. By removing supports and adding others in the nearby non-critical area, one can maintain the print stability and avoid the misconfiguration of fine geometry where needed. In case problems such as friction between the lid and chamber arise, they can be fixed by simple filing using a triangular file.

### Labeling

To enhance the label readability, non-drying modeling clay (Ultra blue, 10106, Van Aaken) was used to fill the lettering grooves (Fig 4A). Remaining clay debris was removed by simple smudging action with Kimwipes. The modeling clay in the grooves was fixed by a thin layer of superglue.

### *In Situ* Hybridization

*In situ* hybridization was conducted according to the previously published protocol [18]. Col2a1 antisense riboprobe was made by RT-PCR and *in vitro* transcription using the following primers: forward primer 5’ GTTGGACCTGCTGGTAAGGA 3’, T7 reverse primer 5’ TAATACGACTCACTATAGGGAGATTCTTGCGTGGGATTTTAGG 3’. L-plastin antisense riboprobe was made by T7 RNA polymerase reaction after a full-length L-plastin IMAGE clone 6794749 was digested with EcoRI.

### Zebrafish Care

The AB* wildtype zebrafish were housed in automatic fish housing systems (Aquaneering, San Diego). All zebrafish procedures were approved by North Carolina Central University Insitutional Animal Care and Use Committee.

## Supporting Information

S1 File 6 hole chambers A-F. 6 hole Chamber A for Walled.stl. 6 hole Chamber B for Walled.stl. 6 hole Chamber C for Walled.stl. 6 hole Chamber D for Walled.stl. 6 hole Chamber E for Walled.stl. 6 hole Chamber F for Walled.stl.
(ZIP)

S2 File 3 hole chambers A-F. 3 hole Chamber A for Walled.stl. 3 hole Chamber B for Walled.stl. 3 hole Chamber C for Walled.stl. 3 hole Chamber D for Walled.stl. 3 hole Chamber E for Walled.stl. 3 hole Chamber F for Walled.stl.
(ZIP)

S3 File Lids A-F for 6 hole chambers. Walled 6 hole Lid A.stl. Walled 6 hole Lid B.stl. Walled 6 hole Lid C.stl. Walled 6 hole Lid D.stl. Walled 6 hole Lid E.stl. Walled 6 hole Lid F.stl.
(ZIP)

S4 File Lids A-F for 3 hole chambers. Walled 3 hole Lid A.stl. Walled 3 hole Lid B.stl. Walled 3 hole Lid C.stl. Walled 3 hole Lid D.stl. Walled 3 hole Lid E.stl. Walled 3 hole Lid F.stl.
(ZIP)

S5 File Lifter 1mm wider. Lifter 1mm wider.stl.
(ZIP)
